# Development of a Multiplex RT-PCR Detection for Six Viruses Infecting Strawberry

**DOI:** 10.3390/v16121858

**Published:** 2024-11-29

**Authors:** Yong Wang, Xiangguo Zeng, Guilin Xiao, Dongmei Zhang, Xin Wen, Xinxin Zhou, Zexian Wang, Jiangli Deng, Yongchao Han

**Affiliations:** Institute of Industrial Crops, Hubei Academy of Agricultural Sciences, Wuhan 430064, China; 13429883936@163.com (Y.W.); xiangguozeng1978@163.com (X.Z.); xgl@hbaas.com (G.X.); zhang_dm321@163.com (D.Z.); 13706873959@163.com (X.W.); zhouxx0905@163.com (X.Z.); cmjasonw@163.com (Z.W.); 15871338530@163.com (J.D.)

**Keywords:** strawberry, multiplex RT-PCR, SMYEV, SVBV, SMoV, SPV-1, SPaV, SCrV-4

## Abstract

Strawberry viruses are significant pathogenic agents in strawberry. The development and application of efficient virus detection technology can effectively reduce the economic losses incurred by virus diseases for strawberry cultivators. In order to rapidly identify strawberry virus species and prevent the spread of virus disease, a multiplex reverse transcription polymerase chain reaction system was established for the simultaneous detection and identification of strawberry mild yellow edge virus (SMYEV), strawberry vein banding virus (SVBV), strawberry mottle virus (SMoV), strawberry polerovirus 1 (SPV-1), strawberry pallidosis-associated virus (SPaV), and strawberry crinivirus 4 (SCrV-4). In this study, six pairs of specific primers were designed on the conserved genomic regions of these viruses. The primer concentration, annealing temperature, and amplification cycle number of the reaction system were optimized. Subsequent sensitivity testing and application of the optimized detection system were carried out. The results indicate the establishment of an efficient detection system for strawberry viruses. The optimal reaction can detect the six viruses at the same time, which provides technical support for the early prevention and treatment of strawberry virus diseases.

## 1. Introduction

Strawberry is a valuable small fruit crop with high economic significance, cultivated extensively across the world. Global strawberry production reached USD 14 billion in 2020, with China being the world’s largest producer, generating USD 5 billion in output [1,2]. However, multiple pathogens and insects pose significant threats to strawberry plants, especially viruses. Strawberry viruses are difficult to observe directly and can be transmitted through multiple pathways, including insect vectors. For instance, the aphid (*Chaetosiphon fragaefolii*) and whitefly (*Trialeurodes vaporariorum*) are the main insect vectors for strawberry virus transmission [3,4]. This makes strawberry virus disease difficult to control. Strawberry viruses often have the phenomenon of multiple infection in the field, leading to severe disease. Single and multiple infections of these viruses can cause malformation of strawberry plants, reduced yields, and deterioration of fruit quality, leading to severe economic losses [5,6,7], posing a significant threat to the strawberry industry. To minimize the losses caused by viral diseases, it is crucial to ensure the virus-free status of seedlings in strawberry cultivation through stringent testing.

At least 30 different viruses are known to infect strawberries globally [3,8]. Among them, strawberry vein banding virus (SVBV), belonging to the genus *Caulimovirus*, has a circular and double-stranded DNA genome [9,10]. Its impacts on strawberry ranged from almost asymptomatic latent infections to necrosis and serious dwarfing of the whole plant. Infected strawberry plants show signs of leaf yellowing, significant reduction in stolons, and growth retardation, which eventually leads to a significant decrease in yield and quality. These symptoms are particularly pronounced when mixed with other viruses [5,11,12]. Strawberry mild yellow edge virus (SMYEV) has a positive-sense single-stranded RNA genome encased in a filamentous viral particle and belongs to the genus *Potexvirus*. It was first characterized in 1990 [13]. Strawberry plants infected by SMYEV alone usually have no obvious symptoms, but one research study found that asymptomatic strawberry plants infected by SMYEV showed a reduction ranging between 28% and 63% in total and marketable fruit number and weight [7]. Strawberry mottle virus (SMoV) has a bipartite RNA genome and belongs to the genus *Sadwavirus*. It can lead to yield losses of up to 30% from single infection. The losses can be higher if coinfected with SVBV and/or SMYEV [14]. Strawberry polerovirus 1 (SPV-1) has an RNA genome and belongs to the genus *Polerovirus*. It was first detected in diseased strawberry plants with decline symptoms in eastern Canada in 2015 [6]. This virus has since been reported in several countries, but has not yet been documented in China. Strawberry pallidosis-associated virus (SPaV) has an RNA genome, belongs to the genus *Crinivirus*, and is associated with strawberry pallidosis disease [4,15]. Strawberry crinivirus 4 (SCrV-4) has an RNA genome and belongs to the genus *Crinivirus*. It is reported to be associated with strawberry decline, and has been detected in Canada, the USA, and Iran [16,17,18].

Currently, several methods, including ELISA [19], simplex RT-PCR, uniplex real-time RT-PCR [20], and the reverse transcription loop-mediated isothermal amplification (RT-LAMP) or LAMP [5,21], have been developed for the detection of strawberry viruses. However, these approaches allow detection for a single virus only. As the number of viruses requiring testing increases, the associated costs and workload will also increase. Multiplex RT-PCR amplifying multiple nucleic acid fragments in one reaction enables rapid and sensitive identification of several viruses simultaneously in a single assay, which greatly reduces cost and increases efficiency of viral surveys. To date, multiplex RT-PCR has been used widely to detect viruses in potato [22], kiwifruit [23], lily [24], soybean [25], and tobacco [26]. Although multiplex RT-PCR has been reported for the detection of viruses in strawberry [27], simultaneous detection of six different strawberry viruses is rarely documented.

In this study, specific primer pairs were designed for SMYEV, SVBV, SMoV, SPV-1, SPaV, and SCrV-4. After optimizing the reaction conditions, an efficient multiplex RT-PCR method capable of simultaneously detecting the six viruses infecting strawberries was developed. This method provides an economically efficient approach for field virus detection in strawberries and rapid quality control of strawberry virus-free seedlings.

## 2. Materials and Methods

### 2.1. Plant Materials and the Positive RNA Sample

Fresh leaves from strawberry grown in our germplasm resource nursery, which were confirmed to be infected with SMYEV, SVBV, SMoV, SPV-1, SPaV, and SCrV-4 by RT-PCR and sequencing, were used to establish and optimize the multiplex RT-PCR assay. We mixed the RNA containing different viruses to create a positive sample harboring the 6 viruses. This positive sample was utilized for both the uniplex and multiplex RT-PCR.

### 2.2. RNA Extraction and Reverse Transcription

Total RNA was extracted using the HiPure Plant RNA Mini Kit (Magen Biotechnology Co., Ltd., Guangzhou, China). Briefly, after the fresh strawberry leaves were quickly ground into powder with liquid nitrogen, 100 mg powder was added to 800 μL PRC1 buffer, thoroughly mixed, left for 5 min, centrifuged at 14,000× *g* for 5 min. A total of 700 μL of supernatant was taken for subsequent column adsorption and elution, and finally, the purified total RNA was eluted with 50 μL RNase-free water. To check the quality of total RNA, 4 μL of total RNA was taken for agarose gel electrophoresis. The first strand of cDNA was synthesized with the ToloScript All-in-one RT EasyMix for qPCR (Tolo Biotech., Shanghai, China) according to the manufacturer’s protocol. Briefly, the 20 μL reverse transcription mixture contained 4 μL of 5× All-in-one RT buffer, 1 μL of All-in-one Enzyme Mix, 4 μL of template RNA, and RNase-free ddH_2_O. Then, the mixture was incubated at 25 °C for 5 min, followed by incubation at 60 °C for 15 min, and finally, the enzyme was inactivated at 85 °C for 5 s.

### 2.3. Design of Virus-Specific Primers

The genomic sequences of SMYEV (accession numbers: KR350471.1, KR559736.1, and KR707814.1), SVBV (accession numbers: MF197916.1, HE681085.1, and MZ328104.1), SMoV (accession numbers: MW383502.1, MT991103.1, MT991101.1, MT070755.1, MT070753.1, and MT070752.1), SPV-1 (accession numbers: KM233705.1, KM233706.1, MZ328110.1, MZ328111.1, and MZ351169.1), SPaV (accession numbers: AY488138.2, MN747002.1, MZ328106.1, MZ328109.1, MZ351164.1, and OP839188.1), and SCrV-4 (accession numbers: MZ326666.1, MZ868643.1, EU490423.1, KU237245.1, and KY488557.1) were retrieved from GenBank. Nucleotide sequence alignment was performed using the software DNAMAN (Version 8, Lynnon Corporation, Vaudreuil-Dorion, QC, Canada) to identify conserved regions. Virus-specific primers were designed based on the identified conserved regions identified for each virus using Primer Premier 5.0 software (Premier Bio-soft International, Palo Alto, CA, USA). These primers (Table 1) were synthesized by AuGCT Biotech in Wuhan, Hubei province.

### 2.4. Evaluation of Primer Specificity

The cDNA of the positive sample was used as the PCR template. Each pair of primers was employed to detect the positive sample in uniplex PCR. Each 20 μL reaction mixture contained 10 μL 2×EsTaq Mix, 0.4 μL of each primer (10 μM), 1 μL cDNA and 8.2 μL double-distilled water. The thermal cycling conditions were as follows: 95 °C for 3 min, followed by 35 cycles of 94 °C for 30 s, 60 °C for 45 s, and 72 °C for 1 min, with a final extension at 72 °C for 5 min. The PCR products were electrophoresed in a 2% agarose gel using 1×TAE buffer.

To further confirm the specificity of each primer pair, the PCR products were cloned into the pMD19-T vector (Takara) for sequencing. Sequence alignment was performed using DNAMAN software.

### 2.5. Optimization of Multiplex RT-PCR Assay

The multiplex RT-PCR was optimized by adjusting the primer concentrations, annealing temperature, and the number of amplification cycles. The primer concentrations are listed in Table 2. Different annealing temperatures were set from 54.0 °C to 66.0 °C. The number of amplification cycles ranged from 25 to 40 (25; 30; 35; 40). The multiplex RT-PCR mixture contained 10 μL 2×EsTaq Mix and 1 μL cDNA of the positive sample; the final concentrations of the six pairs of primers are listed in Table 2, with the volume adjusted to 20 μL using the double-diluted water.

### 2.6. Sensitivity of Multiplex RT-PCR Assay

To compare the sensitivity of multiplex RT-PCR with that of uniplex RT-PCR, 10-fold serial dilutions of cDNA from the positive sample containing SMYEV, SVBV, SMoV, SPV-1, SPaV, and SCrV-4 were used as templates. Except for the difference in the addition of primers, the amplification conditions of the uniplex RT-PCR and multiplex RT-PCR were exactly the same as the conditions optimized above. The PCR products were subjected to electrophoresis for analysis.

### 2.7. Survey of Strawberry Viruses Using Multiplex RT-PCR Assay

A total of 37 strawberry plants with clear virus infection information were selected from our experimental base for the detection of the six viruses using the optimized multiplex RT-PCR assay. Among these, 22 samples were of the cultivar ‘Benihoppe’, while the remaining 15 samples were ‘Kaorino’. The RNA extraction and cDNA synthesis were performed following the procedure described above. The PCR products were analyzed as previously described.

## 3. Results

### 3.1. Specificity of Primer Pairs

**Figure 1:**
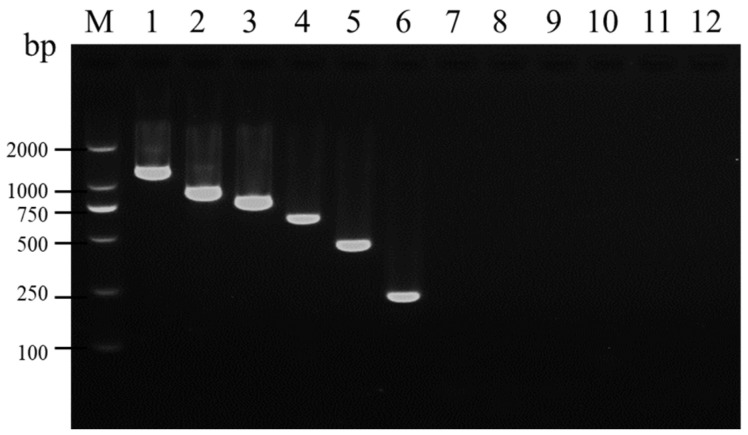
Determination of specificity of the six primer pairs used in uniplex RT-PCR. Lane M: TL-2000 DNA marker; lanes 1–6: detection of SMYEV, SVBV, SMoV, SPV-1, SPaV, and SCrV-4 using the viral-specific primer pairs with a template containing the six viruses, respectively; lanes 7–12: detection of SMYEV, SVBV, SMoV, SPV-1, SPaV, and SCrV-4 using the viral-specific primer pairs with a template that does not contain any of the six viruses, respectively.

In the uniplex RT-PCR, fragments of the expected sizes for SMYEV, SVBV, SMoV, SPV-1, SPaV, and SCrV-4 were specifically amplified using viral-specific primer pairs (Figure 1). To further validate the specificity of the designed primer pairs, the amplified fragments were individually cloned into the pMD19-T vector and subsequently sequenced. After sequencing, alignment using BLASTn showed over 96% consistency between the amplified products and the corresponding sequences of SMYEV, SVBV, SMoV, SPV-1, SPaV, and SCrV-4 in NCBI. This indicates that these primers can specifically detect the corresponding target viruses.

### 3.2. Optimization of Multiplex RT-PCR Assay

**Figure 2:**
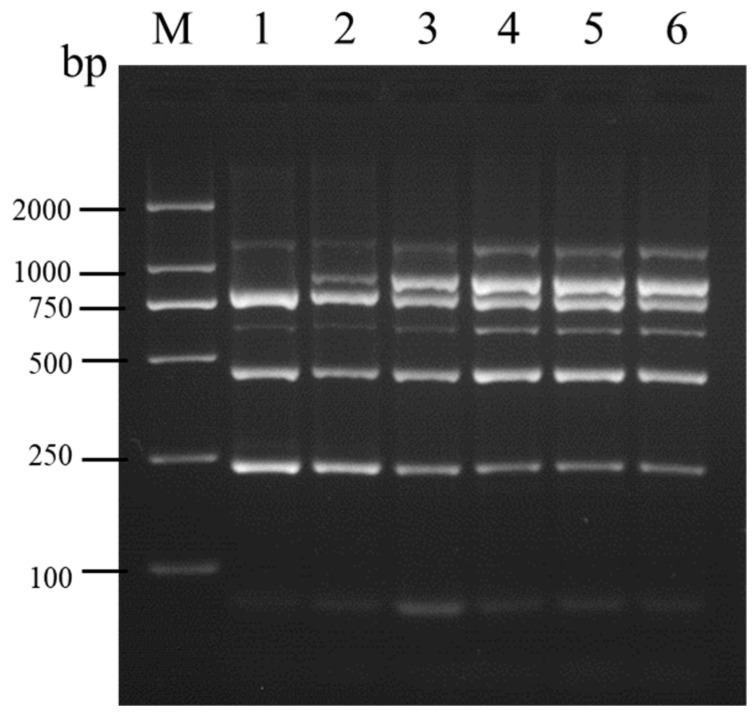
Optimization of primer concentrations for multiplex RT-PCR aimed at detecting SMYEV, SVBV, SMoV, SPV-1, SPaV, and SCrV-4. Lane M: TL-2000 DNA marker; lanes 1–6: groups 1–6 corresponding to different primer concentrations.

Following multiplex RT-PCR with various combinations of primer concentrations, clear amplification bands for six viruses were achieved with the primer concentrations in groups 4, 5, and 6, in comparison to the other combinations (Figure 2). To reduce primer usage, the primer concentration combination from group 4 was selected for further optimization of the multiplex RT-PCR conditions.

**Figure 3:**
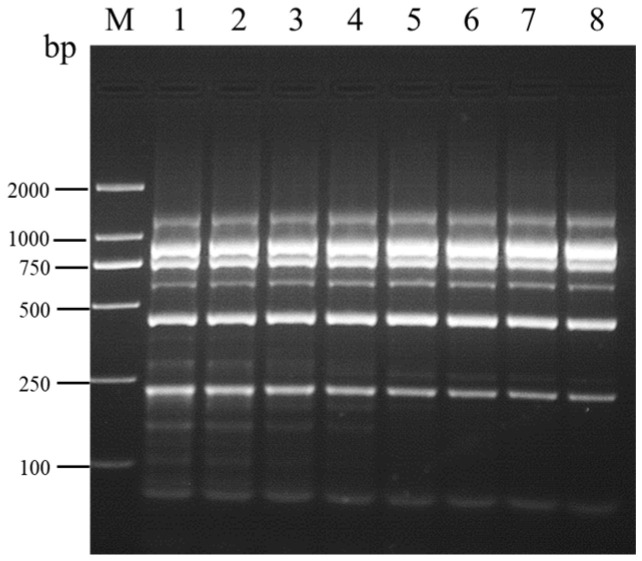
Optimization of annealing temperature for the multiplex RT-PCR assay detecting SMYEV, SVBV, SMoV, SPV-1, SPaV, and SCrV-4. Lane M: TL-2000 DNA marker; lanes 1–8: 54.0 °C; 54.7 °C; 55.9 °C; 57.8 °C; 60.1 °C; 62.0 °C; 63.2 °C; 64.0 °C. The amplification cycle number for this assay was set to 35 cycles.

Following multiplex RT-PCR at eight different annealing temperatures, amplification was observed for all six viruses. Faint non-specific bands appeared at annealing temperatures ranging from 54.0 °C to 57.8 °C, while the presence of non-specific bands significantly decreased at annealing temperatures above 60.1 °C, albeit with residual faint bands (Figure 3). In light of energy consumption considerations, an annealing temperature of 60.1 °C was selected for subsequent optimization.

**Figure 4:**
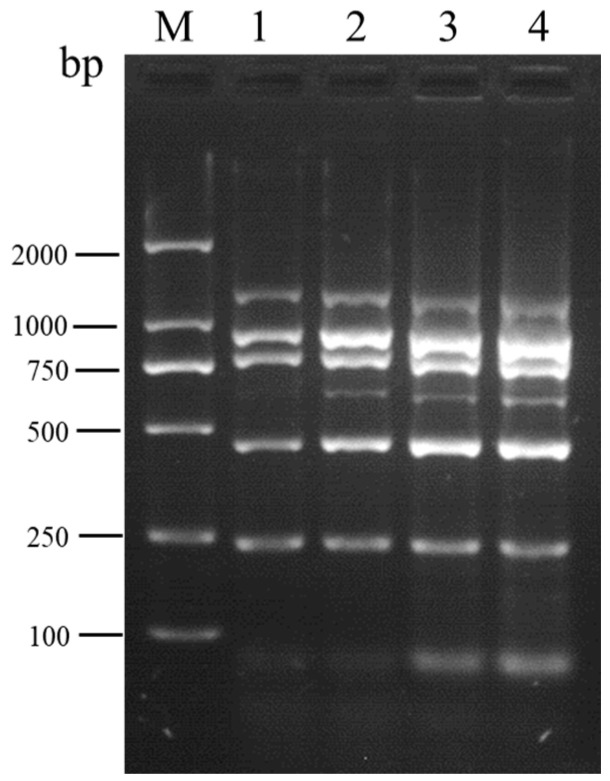
Optimization of the amplification cycle number for multiplex RT-PCR designed for the simultaneous detection of SMYEV, SVBV, SMoV, SPV-1, SPaV, and SCrV-4. Lane M: TL-2000 DNA marker; lanes 1–4: 25 cycles; 30 cycles; 35 cycles; 40 cycles, respectively.

To mitigate the occurrence of non-specific bands in multiplex RT-PCR, four different amplification cycle numbers (25, 30, 35, and 40) were tested. Electrophoresis analysis revealed the absence of non-specific bands in the amplification products at 25 and 30 cycles. Notably, when compared to the 25-cycle amplification, the detection of SPV-1 was markedly enhanced at 30 cycles (Figure 4). Consequently, the amplification cycle number was determined to be optimally set at 30.

Based on the aforementioned results, in the optimized multiplex RT-PCR reaction system, the dosage of 2×EsTaq Mix was 10 μL, the final concentrations of the forward and reverse primers of SMYEV were 0.3 μmol/L, those of SVBV were 0.3 μmol/L, those of SMoV were 0.075 μmol/L, those of SPV-1 were 0.4 μmol/L, those of SPaV were 0.25 μmol/L, those of SCrV-4 were 0.075 μmol/L, ddH_2_O was 3.4 μL, cDNA template was 1 μL, and the total volume was 20 μL. The multiplex PCR procedure annealing temperature was 60.1 °C for 45 s, and the cycle number was 30.

### 3.3. Sensitivities of Uniplex RT-PCR and Multiplex RT-PCR Assays

**Figure 5:**
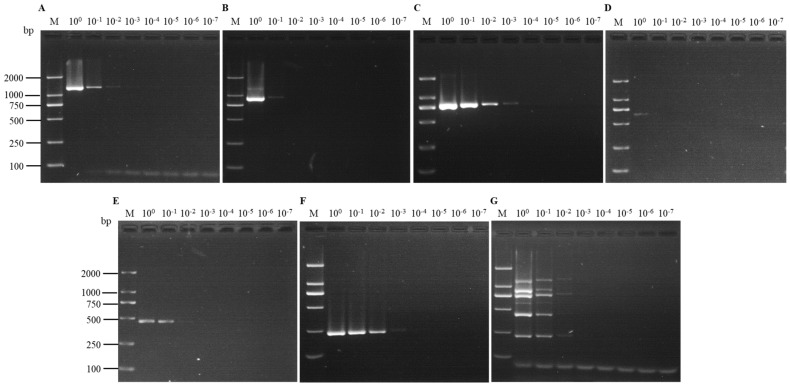
Comparison of the sensitivities of uniplex RT-PCR assays for the detection of SMYEV (**A**), SVBV (**B**), SMoV (**C**), SPV-1 (**D**), SPaV (**E**), and SCrV-4 (**F**), as well as the multiplex RT-PCR assay (**G**). Lane M: TL-2000 DNA marker; lanes 10^0^–10^−7^: ten-fold serial dilutions of the template containing the six viruses.

The sensitivity of each assay was assessed using 10-fold serial dilutions (10^0^–10^−7^) of cDNA derived from the positive sample containing the six viruses. According to the electrophoresis, the uniplex RT-PCR was able to detect SMoV in the cDNA diluted up to 10^−3^, SCrV-4 in cDNA diluted up to 10^−2^, SMYEV, SVBV and SPaV in cDNA diluted up to 10^−1^, and SPV-1 from the original cDNA. For the multiplex RT-PCR, all viruses could be detected in the cDNA diluted up to 10^−1^, except for SPV-1, which could be detected from the original cDNA (Figure 5).

### 3.4. Application of Multiplex RT-PCR Assay in Survey of Strawberry Viruses

**Figure 6:**
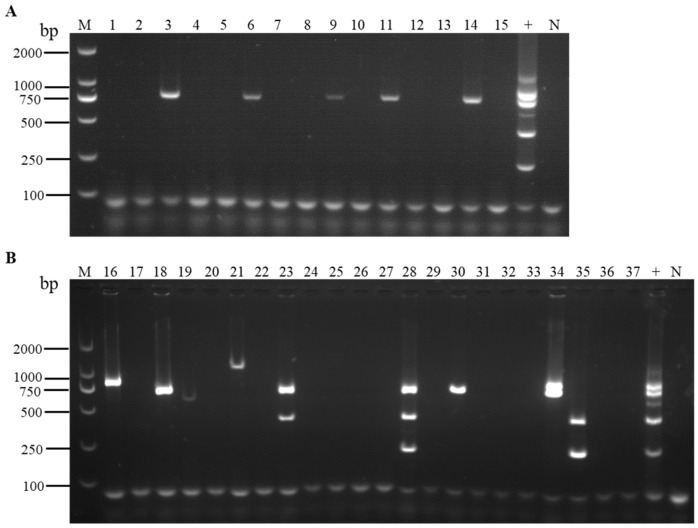
Detection results of field strawberry samples from cultivars ‘Kaorino’ (**A**) and ‘Benihoppe’ (**B**), analyzed using the optimized multiplex RT-PCR, respectively. Lanes 1–15: samples of the cultivar ‘Kaorino’; lanes 16–37: samples of the cultivar ‘Benihoppe’; lane +: the positive sample containing the six viruses; lane N: the negative control; lane M: TL-2000 DNA marker.

We used the optimized multiplex RT-PCR system to detect the six viruses in field strawberry samples. The results indicated that out of 37 samples, 14 were infected by viruses (37.84%). Among these, ten samples were infected by a single virus (27.03%), three samples were infected by two viruses (8.11%), and one sample was infected by three viruses (2.70%) (Figure 6).

## 4. Discussion

When propagating strawberries through vegetative means over the long term, viruses can be transmitted via stolons and insect vectors, resulting in more severe viral diseases affecting strawberries. Among the six viruses, viruses SPaV and SCrV-4 have been reported as new strawberry viruses in China in the past few years, while SPV-1 has not yet been reported domestically. It is the first report of SPV-1 in China. The geography distribution of these three viruses needs further investigation in China.

In multiplex RT-PCR reactions, the design of primers is crucial. It is essential to ensure the specificity of the primers while also optimizing the size of the amplicons for clear differentiation of the corresponding viral fragments during agarose gel electrophoresis. In this study, the size differences of the amplicons corresponding to each virus were greater than 100 bp, which facilitated their separation through agarose gel electrophoresis. To obtain clear detection results, high-concentration agarose gels should be employed for electrophoresis. In this study, a 2% agarose gel was utilized, which is consistent with concentrations employed in other similar studies [28,29]. This is due to the fact that lower-concentration agarose gels tend to cause band dispersion during electrophoresis, complicating the differentiation of bands with similar sizes.

Multiplex PCR/RT-PCR has been used in a variety of scenarios, and the number of targets detected in a single reaction is the focus of this technique. It has been reported that an optimized multiplex RT-PCR can simultaneously detect 25 genes in a single reaction tube [30]. By optimizing the reaction conditions, the GeXP-multiplex RT-PCR can simultaneously detect eight subtypes of influenza A virus [31]. However, for plant viruses, the number of target viruses detected by most multiplex RT-PCR detection systems is less than six [25,32,33]. In this study, we tried to add the specific primers for strawberry crinivirus 3 to the optimized amplification system to establish a system to detect seven viruses simultaneously, but failed. It may be due to the intense competition between primers. The detection system we established has a good advantage in the number of target viruses. Its popularization and application will save the workload of identification of virus-free strawberry seedlings and reduce the production cost.

In this study, the sensitivity of multiplex RT-PCR was found to be lower than that of uniplex RT-PCR in the detection of SMoV and SCrV-4. This may be due to the low concentrations of primers for these two viruses in the multiplex RT-PCR system. To obtain a full validation of the test, more experiments should be conducted to ensure its exclusivity, repeatability, reproducibility, and robustness. For exclusivity testing, more viruses phylogenetically close to the target virus should be added for testing if possible. For repeatability testing, multiple tests on a set of samples under the same laboratory conditions, using the same equipment and reagents, should be conducted. For reproducibility testing, tests on the same set of samples in different laboratory settings, with different operators using the same testing method, should be performed. Finally, minor alterations to the test conditions should be made to assess the impact of the changes on the test results, thereby determining the robustness of the detection method. Awaiting complete validation, this method may already contribute to the development of a healthy strawberry industry and the epidemiological study of strawberry viruses.

## 5. Conclusions

In this study, the optimal combination of primer concentrations, annealing temperature, and cycle number was selected to establish an effective multiplex RT-PCR system for detecting six strawberry viruses in a single reaction. Under the premise of ensuring accuracy, the method can greatly reduce the workload of virus detection of strawberry seedlings, and it is expected to be popularized and applied in production.

## Figures and Tables

**Table 1:** Primers for uniplex and multiplex RT-PCR assays.

Virus	Primer	Sequence (5′-3′)	Product Size (bp)	Melting Temperature (°C)
SMYEV	Multi-SMYEV-F1	TGGCGGATTCCAATACTTGCC	1263	60
	Multi-SMYEV-R1	CTCATCCAGTCTTGGAGGCG		60
SVBV	Multi-SVBV-F	TTGAAGCCTTCGCAGAAGCC	931	60
	Multi-SVBV-R	AGCTTGAGTAGCTCTTCGATCTGAG		60
SMoV	Multi-SMoV-F	CGTGAGAGGACCCGAGCTTATG	792	62
	Multi-SMoV-R	AGAGATAGTCGAACCTTTGGTGGTC		60
SPV-1	Multi-SPV1-F	AACGCGGAGTGGAGAGTGAC	635	61
	Multi-SPV1-R	CATTTGTTCCGCCGCTTCTC		59
SPaV	Multi-SPaV-F	CCATGGAGAATGTGGAGAGCAG	449	59
	Multi-SPaV-R	CACCACGTTCTCTTCCTCAGG		59
SCrV-4	Multi-SCrV4-F	ATAGGCGCGAAATCCAAACTTCC	230	60
	Multi-SCrV4-R	TCAATGATAGGTTCGCTGTTCGC		60

**Table 2:** Different groups of primer sets for multiplex RT-PCR.

Viral Primer	Final Primer Concentrations (μmol/L)					
	1	2	3	4	5	6
Multi-SMYEV-F1/R1	0.2	0.3	0.3	0.3	0.4	0.4
Multi-SVBV-F/R	0.2	0.15	0.3	0.3	0.3	0.3
Multi-SMoV-F/R	0.2	0.15	0.1	0.075	0.075	0.075
Multi-SPV1-F/R	0.2	0.3	0.3	0.4	0.4	0.4
Multi-SPaV-F/R	0.2	0.2	0.25	0.25	0.25	0.2
Multi-SCrV4-F/R	0.2	0.2	0.1	0.075	0.075	0.075

## Data Availability

The data supporting the findings are included in this study, no new data were created.
